# An Expedited Route to Optical and Electronic Properties at Finite Temperature via Unsupervised Learning

**DOI:** 10.3390/molecules28083411

**Published:** 2023-04-12

**Authors:** Fulvio Perrella, Federico Coppola, Nadia Rega, Alessio Petrone

**Affiliations:** 1 Scuola Superiore Meridionale, Largo San Marcellino 10, I-80138 Napoli, Italy; fulvio.perrella-ssm@unina.it (F.P.); federico.coppola@unina.it (F.C.); nadia.rega@unina.it (N.R.); 2 Department of Chemical Sciences, University of Napoli Federico II, Complesso Universitario di M.S. Angelo, via Cintia 21, I-80126 Napoli, Italy; 3 Istituto Nazionale di Fisica Nucleare, Sezione di Napoli, Complesso Universitario di M.S. Angelo ed. 6, via Cintia 21, I-80126 Napoli, Italy

**Keywords:** density functional theory, machine learning, computations of optical spectra, molecular dynamics, clustering techniques

## Abstract

Electronic properties and absorption spectra are the grounds to investigate molecular electronic states and their interactions with the environment. Modeling and computations are required for the molecular understanding and design strategies of photo-active materials and sensors. However, the interpretation of such properties demands expensive computations and dealing with the interplay of electronic excited states with the conformational freedom of the chromophores in complex matrices (i.e., solvents, biomolecules, crystals) at finite temperature. Computational protocols combining time dependent density functional theory and ab initio molecular dynamics (MD) have become very powerful in this field, although they require still a large number of computations for a detailed reproduction of electronic properties, such as band shapes. Besides the ongoing research in more traditional computational chemistry fields, data analysis and machine learning methods have been increasingly employed as complementary approaches for efficient data exploration, prediction and model development, starting from the data resulting from MD simulations and electronic structure calculations. In this work, dataset reduction capabilities by unsupervised clustering techniques applied to MD trajectories are proposed and tested for the ab initio modeling of electronic absorption spectra of two challenging case studies: a non-covalent charge-transfer dimer and a ruthenium complex in solution at room temperature. The K-medoids clustering technique is applied and is proven to be able to reduce by ∼100 times the total cost of excited state calculations on an MD sampling with no loss in the accuracy and it also provides an easier understanding of the representative structures (medoids) to be analyzed on the molecular scale.

## 1. Introduction

Photo-induced phenomena and optical properties are the grounds to investigate electronic states and their interactions with the environment [1,2,3,4,5,6,7,8,9,10,11,12,13,14,15,16,17,18,19,20,21,22]. Experimental spectra can be interpreted via computational approaches at the molecular scale, understanding the microscopic characteristics that determine the position, width and shape of absorption bands [23,24,25,26,27,28,29,30,31,32,33,34]. However, a number of challenges remain open and mainly concern the modeling of either floppy molecules or non-covalent complexes in solution. Ideal approaches to deal with the complexity of the conformational freedom have to ensure an adequate sampling of the phase space of the potential energy surface (PES) at a given temperature, since such systems cannot be easily described by minimum energy structures as starting points for subsequent more computationally expensive calculations required to compute electronic transitions and excited state properties [34,35,36]. Molecular dynamics (MD) is the perfect technique for this goal since it can simultaneously describe the conformational freedom and the complexity of the environment (i.e., explicit solvent models) and can guarantee a satisfactory sampling of the phase space of the PES for selecting the initial states of the electronic transitions. On these bases, it is possible to reproduce the thermal fluctuations in a classical manner and simulate the shape of the electronic spectrum by classically considering the spreading of the vertical transitions of a representative sample of snapshots of the MD trajectory [37,38,39].

An accurate description of the electronic layout of a system is very important for the excited state properties and optical absorption. The electronic state separation, and the resulting UV-Vis absorption, strongly depend on the reference structure(s), indeed. A very detailed description of the PES ruling the system dynamics is demanded when standard force fields cannot by easily used, i.e., with non-covalent charge-transfer complexes [40,41,42,43,44,45,46,47,48], metal compounds or usually when the electronic density reorganization is involved during the time, even in the ground state. This is usually quite common also when an environment reorganization is involved as well. Since parameterized force fields cannot account for explicit electronic effects, an explicit treatment of electronic degrees of freedom is mandatory via ab initio methods. However, when reasonable large systems (≥1000 atoms) are studied, accurate wavefunction-based methods cannot be employed due to their high computational cost (above all, for excited state properties), although some progress has been recently achieved using graphical processing units [49] and localization procedures [50,51]. Thus, density functional theory (DFT) and time dependent (TD-) DFT, the latter required for excited state quantities, are usually chosen as a good compromise between accuracy and computational costs [10,52,53,54,55,56,57,58,59,60,61,62].

Besides canonical computational chemistry fields, data analysis and machine learning (ML) methods have been increasingly employed as complementary approaches for an efficient data exploration, prediction and model development, starting from experimental data (structure, properties, reactivity) or from MD simulations and electronic structure calculations [63,64,65,66,67,68,69,70,71,72,73,74,75,76]. In particular, MD simulations often produce very big datasets (i.e., the collected trajectory in the phase-space), especially for long simulation times and extended systems, which can be difficult to manually inspect. Automated ML data analysis techniques thus can offer a valuable and efficient option to extract the significant and “physical” information from MD trajectories. In particular, unsupervised ML methods, such as clustering analysis, are able to partition a dataset according to similarities in some features space, employing only the *input* values and not requiring any *output* ones supplied by the user. Clustering proved a valuable tool for MD simulation analysis, allowing one to reduce the high number of sampled structures into a few representative ones, approximating conformational energy minima [77,78,79,80,81,82,83,84,85,86,87,88,89,90,91,92].

The simulation of electronic band-shapes at finite temperature through an MD sampling potentially requires hundreds of excited state calculations. Therefore, alternative routes such as the selection of a small number of representative frames could both reduce the computational cost of spectra calculations and simplify their interpretation [78,82]. In this work, dataset reduction capabilities via clustering techniques applied to MD trajectories in specifically tailored feature spaces were tested in the simulation of electronic absorption spectra of two model compounds. In particular, spectra computed only from the clusters’ representative frames showed a remarkable reproduction of the main spectral features if compared to spectra from a uniform sampling of frames of the trajectory (a subset of ∼500 structures). This approach also allowed an easier interpretation of the calculated bands, which could result from many states close in energy but differing for their spatial properties.

**Figure 1:**
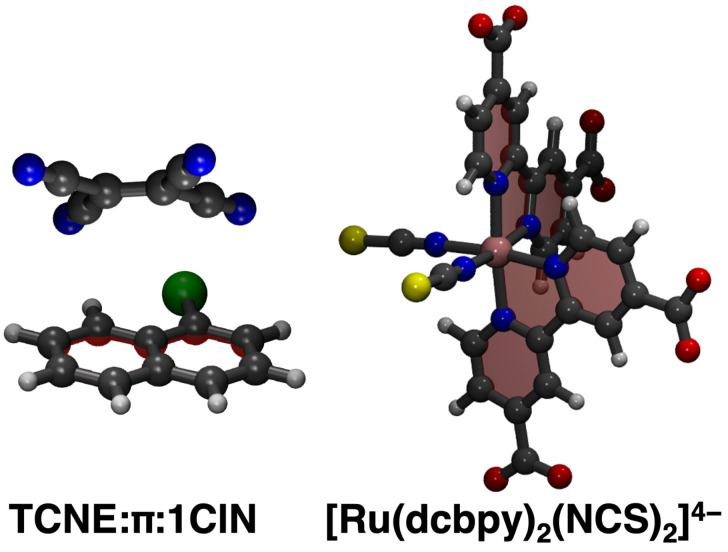
Case studies investigated in the present work. The TCNE:π:1ClN non-covalent dimer and Ru(II) complex ([Ru(dcbpy)_2_(NCS)_2_]^4−^ or “N3^4−^”, dcbpy = 4,4′-dicarboxy-2,2′-bipyridine) are presented from left to right, respectively (Carbon is in gray, Hydrogen in white, Chlorine in green, Sulphur in yellow, Oxygen in red, Nitrogen in blue, Ruthenium in pink).

Dataset reduction capabilities via unsupervised clustering techniques applied to MD trajectories are proposed and tested for the ab initio modeling of electronic absorption spectra of two challenging case studies. The first investigated model system is a prototypical π-stacked non-covalent dimer in dichloromethane (DCM) solvent (see Figure 1, left panel), consisting of an electron donor (1-chloronaphthalene, 1ClN) and an electron acceptor (tetracyanoethylene, TCNE). This represents a challenging case study with respect to evaluating the performance of ML clustering techniques in reproducing its electronic/optical properties, considering that the potential energy surface of weakly bound systems, ruled by dispersive intermolecular forces, is quite flat and numerous isoenergetic orientational isomers can be present in the solution. The TCNE:π:1ClN dimer has been thoroughly investigated in recent years by means of Femtosecond Stimulated Raman Spectroscopy [93] and through electronic structure methods for the detailed characterization of the ground state properties [35] and to unveil the nuclear relaxation upon photoexcitation downhill from the Franck–Condon region of the first charge-transfer state [34,94]. The second system is instead a Ru(II) complex, [Ru(dcbpy)_2_(NCS)_2_]^4−^ (dcbpy = 4,4′-dicarboxy-2,2′-bipyridine) in water solution, also called “N3^4−^” (Figure 1, right panel), which is a popular example of Ru-based dye sensitizers for solar cells and light-harvesting applications [95,96,97]. Light absorption by N3^4−^ in the visible region induced excitation to a dense manifold of metal-to-ligand charge-transfer (^1^MLCT) states. N3^4−^ photo-physical behavior is characterized by an ultrafast relaxation pathway among the singlet and triplet manifolds, induced by a complex interplay between closely spaced coupled electronic states, nuclear motion and solvent rearrangement [98,99,100,101,102,103,104,105], potentially influencing the dynamics and efficiency of the electron injection into a semiconductor substrate [106,107,108,109,110]. The N3^4−^ complex, both for its dense ^1^MLCT manifold and its conformational dynamics in the solution [111], represents therefore another ideal model system for testing an efficient MD/ML clustering approach for the simulation of electronic spectra including finite-temperature effects.

## 2. Results and Discussion

### 2.1. The TCNE:π:1ClN Case Study

**Figure 2:**
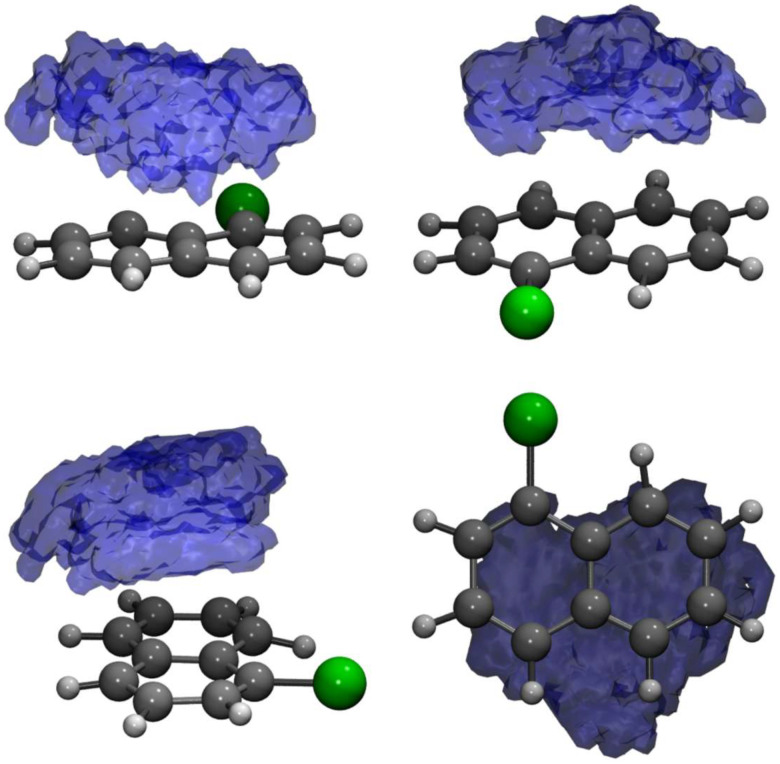
Side, front and top views of the spatial distribution function of the center-of-mass of the TCNE acceptor monomer around the 1ClN subunit.

The massive amount of data acquired during an MD simulation requires analyses that are capable of going beyond the visual inspection of snapshots and average structures. In this regard, the statistical analysis of the trajectories through the calculation of the distribution functions represents an advantageous choice with respect to taking into account conformational dynamics, solute–solvent interactions and so on. In Figure 2, a three-dimensional spatial distribution function (SDF) [112,113] is presented, computed along the 10 ps-long AIMD trajectory by considering the 1ClN as reference molecule (for which a local three points coordinate system was defined) and the center of the mass of the TCNE unit. From the SDF, it is observed that the TCNE remains on the same side of the ClN and slips on both rings during the exploration of the ground state PES, proving the presence of different mutual configurations and distances due to the weak Coulombic interactions that rule the π-stacked arrangement.

**Figure 3:**
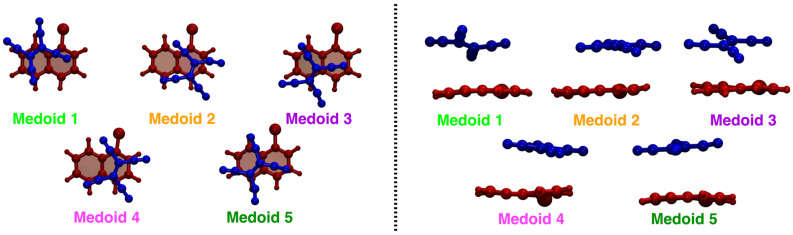
Structures of the five cluster medoids in top (**left** panel) and side (**right** panel) views. The TCNE and 1ClN are represented as ball and stick in blue and red, respectively. The color code is uniform with that of Figure 4.

**Figure 4:**
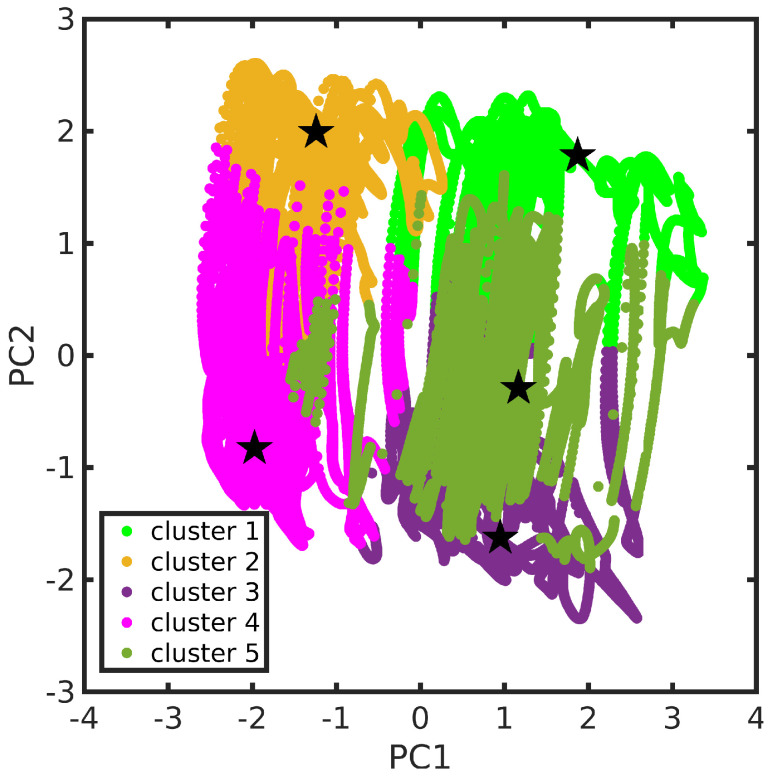
TCNE:π:1ClN trajectory in the features’ first two principal components space. Cluster partition is represented through different colors. Cluster medoids are also highlighted (as star symbols). The color scheme adopted is kept fixed throughout this section.

According to the procedure presented in Section 3.2, the MD trajectory clustering thus yielded, for the TCNE:π:1ClN case study, five medoids (each one is representative of the corresponding cluster), characteristic of the accessible conformational space, in agreement with the SDF previously discussed. The clustering structural feature values shown by the five medoids, namely the rotation angle ($\theta_{r}$) and the rotation axis (${\hat{n}}_{r}$) of the two subunits molecular planes and the position vector between the two geometric centers (${\vec{r}}_{N-E}$), are collected in Table 1 (see also Section 3.2 for features definitions). A detailed analysis of medoid structural features reveals that they do not significantly differ in the rotation angle $\theta_{r}$ between the two TCNE and 1ClN planes (values within a small range, ∼5 degrees), but mainly in the planes’ relative orientation, as suggested by clearly different ${\hat{n}}_{r}$ axis components and secondarily in the TCNE-1ClN relative position. A closer inspection of the rotation axis ${\hat{n}}_{r}$ components for medoids 2 and 4 reveals that they differ along the *x*- and *z*-axes (see Table 1), while for medoids 3 and 5 only the component along the *z*-axis is reoriented for these latter. Additionally, analyzing the five medoids obtained from the clustering approach (side view presented in the right panel of Figure 3), considerable geometric deformations are present and the relative position of the molecular planes is also different. In order to acquire a visual representation of such clustering, the trajectory was projected onto the subspace of the features’ first two principal components (PCs; please refer to Section 3.3 for technical details). A clear cluster separation was obtained, with each medoid representing a different portion of the conformational space (see Figure 4). The partial superposition of cluster 5 with cluster 3 in the principal components subspace is only an artifact (being instead separated in the full space) due to the reduced variance explained by the first two PCs (∼55.5% of the total variance).

From Figure 3, the found medoids overall show pairwise structural similarities (see 2–4 and 3–5) if observed in a top-down direction; see Table 1 for a more quantitative evaluation. The conformational flexibility of the TCNE:1ClN π-stacked complex, which is due to the weak dispersion forces, is thus fully captured with the trajectory clustering approach.

**Figure 5:**
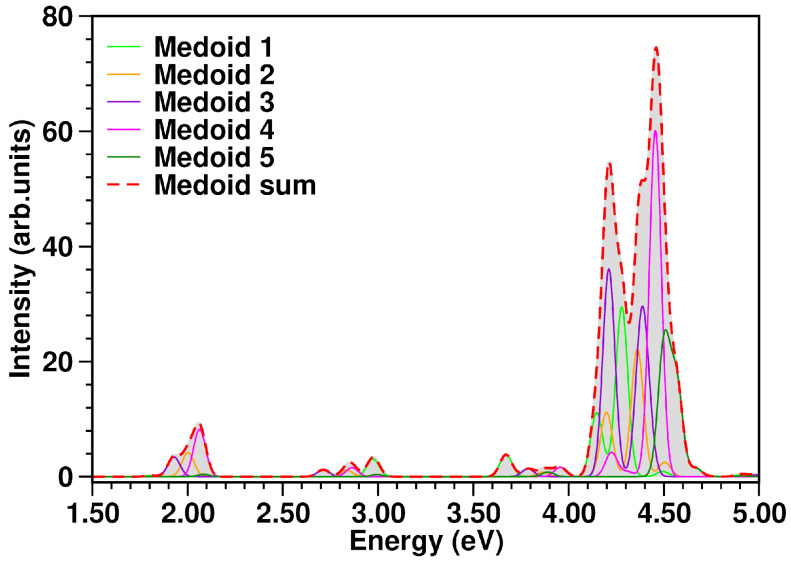
TCNE:π:1ClN absorption spectrum (in eV) calculated at TD-CAM-B3LYP/6-31G(d,p)/GD3/C-PCM(DCM) level of theory from each medoid and as the sum spectrum of the structures representative of the conformational equilibrium in the ground state. The color code is presented in the graph legend. The sum spectrum (red dashed curve) was obtained as the sum of individual medoid contributions (presented in the figure as well, see color legend), each one already multiplied by the k-th cluster population. See Equation (5) and the procedure explained in Section 3.4 for more details.

The UV-Vis spectrum comprising the first low-lying singlet states computed for each medoid within linear response TD-DFT formalism is reported in Figure 5. The estimation of the whole electronic spectrum (red curve) was obtained according to Equation (5) and the procedure explained in Section 3.4. A comprehensive analysis of the electronic spectrum has been recently provided by some of the authors in Ref. [35]. On the other hand, we report a detailed summary of the characterization of the transitions towards the S_1_ and S_2_ excited states in Table 2. We recall that the weaker electronic transitions below 4.00 eV have a charge transfer (CT) nature (for S_1_ and S_2_ see $\omega_{\mathrm{CT}}$ charge transfer descriptor parameter in Table 2). Conversely, the very bright ones are characterized by electronic density reorganization occurring in the same molecular unit, hence they are of a local excitation (LE) character. For medoid 1, the TCNE is located on an edge of the 1ClN ring and it mainly contributes to the absorption bands above 2.50 eV (see light green curve in Figure 5), while for the S_0_–S_1_ electronic transition at 1.807 eV characterized by a strong CT nature ($\omega_{\mathrm{CT}}=0.968$) the probability is negligible, f=0.002. For medoids 2 and 4, the TCNE lies on the ring bearing the chlorine atom and they share roughly the same electronic properties in terms of transition probability and energy range (see orange and magenta curves in Figure 5, respectively). Both show absorption bands in all regions of the spectrum. In this case, the first two states S_1_ and S_2_ of both medoids contribute, respectively, to the bands at ∼2.00 and 2.80 eV. Also in these cases, the S_1_ and S_2_ states are characterized by a strong charge transfer nature as can be easily deduced from the values of the $\omega_{\mathrm{CT}}$ descriptor close to unity, reported in Table 2. In medoids 3 and 5, the TCNE is placed on the unfunctionalized six-membered ring of the 1ClN and we observe that the electronic properties show considerable differences. The electronic features of medoid 3 (violet curve in Figure 5) cover the entire spectral range considered, 1.50–5.00 eV, while only high energy electronic transitions (>3.50 eV) are bright for medoid 5 (dark green curve in Figure 5).

**Figure 6:**
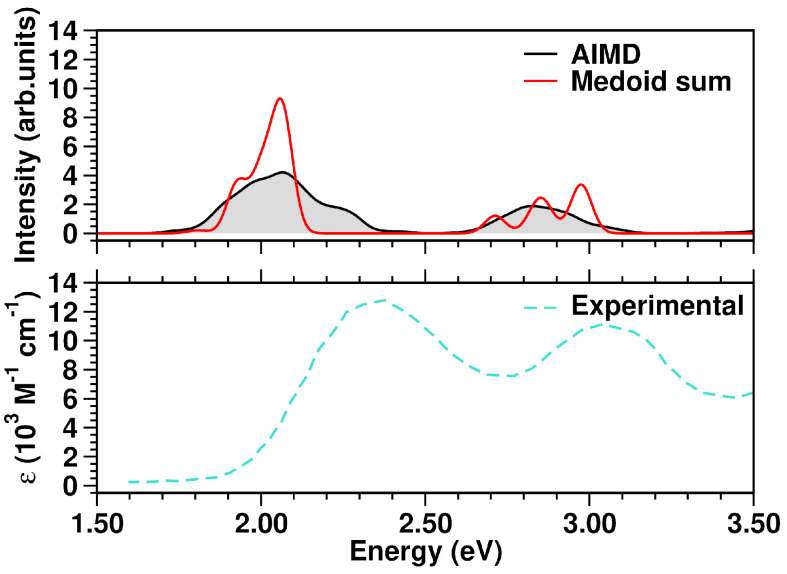
**Top** panel: comparison of TCNE:π:1ClN simulated absorption spectra in the 1.50–3.50 eV range. **Bottom** panel: experimental UV-Vis spectrum, retrieved from Ref. [93], of the TCNE:π:1ClN complex measured in DCM solution (molar absorptivity, ε). The color code is presented in the graph legend.

Comparing the spectrum from the five medoids to that from the complete MD sampling, an excellent agreement is observed (Figure 6, top panel). The experimental optical spectrum profile in solution (Figure 6, bottom panel) shows two distinct absorption bands with maxima centered at 408 nm (3.04 eV) and 537 nm (2.31 eV), as well as the calculated spectrum. In particular, the first calculated band at ∼2.00 eV has contributions from the S_1_ states of representative frames 4, 2 and 3, each having, in turn, a clear 1ClN → TCNE charge-transfer character. Analogously, the second band at ∼2.80 eV appears constituted by the S_2_ CT states of medoids 1, 4, 3 and 2.

Such a case study proves the clustering technique to be an efficient way to estimate the electronic spectrum at finite temperature, avoiding excited state calculations on a large number of frames (for TCNE:π:1ClN model system, a 100-fold decrease in total computational cost). Moreover, the medoid excited state characterization (Table 2) allows one to perform a more accurate spectral assignment of the absorption bands. This further confirms that the cluster medoids, taken as representative frames, can efficiently resume the collected conformational dynamics.

### 2.2. The N3^4−^ Case Study

**Figure 7:**
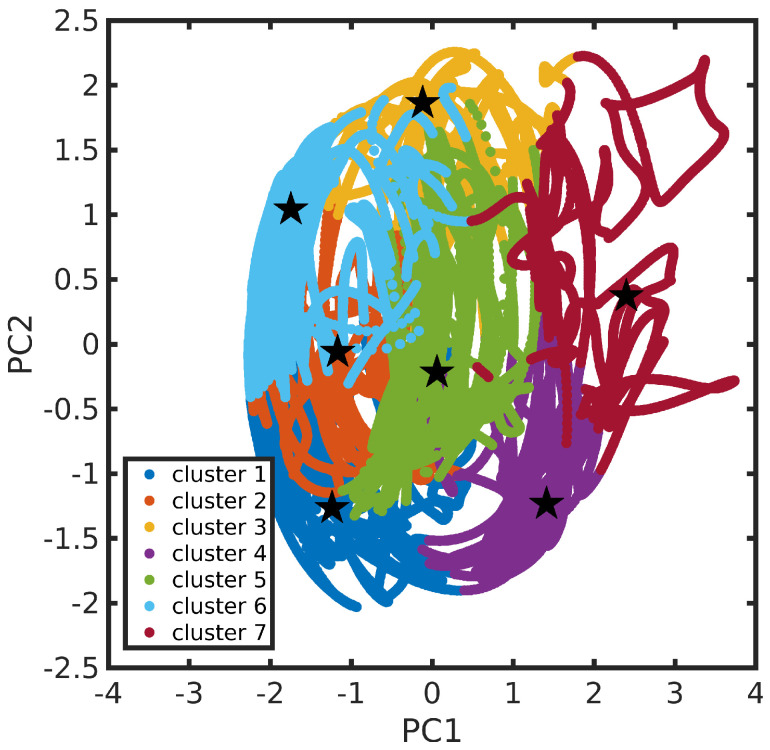
N3^4−^ trajectory in the features’ first two principal components subspace. Cluster partition is represented through different colors. Cluster medoids are also highlighted (as star symbols).

**Figure 8:**
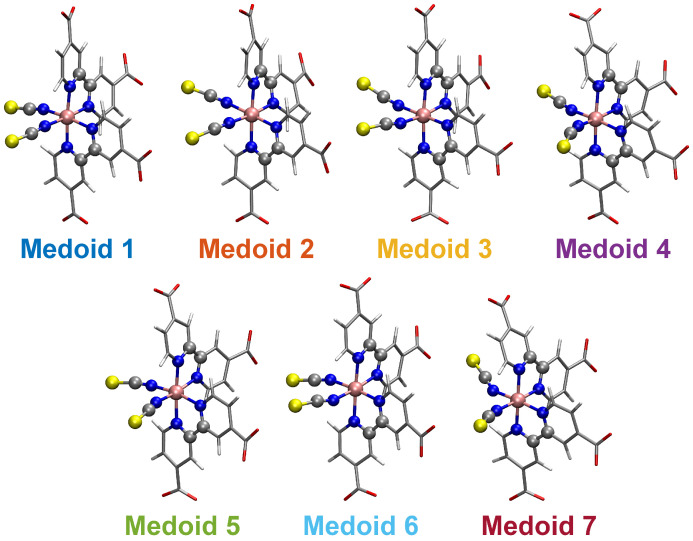
Structures of the N3^4−^ seven cluster medoids. The atoms determining the features employed for clustering analysis are highlighted as ball and stick. The color code is uniform with that of Figure 7.

**Figure 9:**
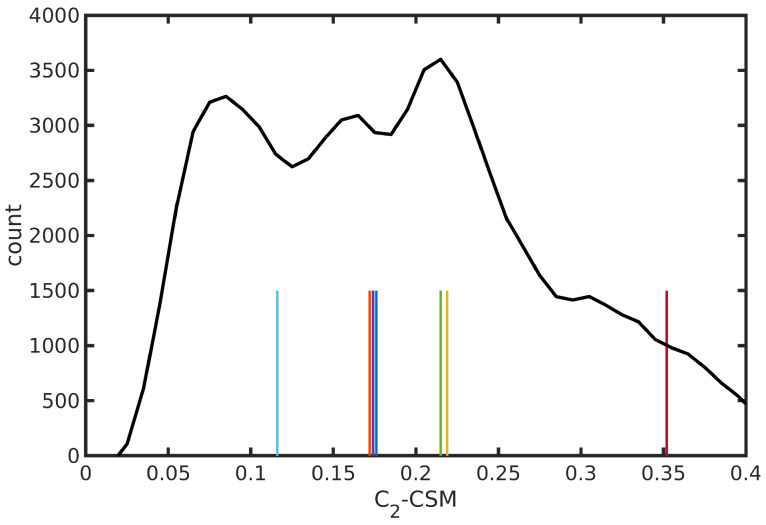
Distribution of $C_{2}$-CSM symmetry deviation parameter from N3^4−^ trajectory in water solution. Values of the medoid structures from trajectory clustering analysis are also shown as vertical bars (with arbitrary heights). The color code is uniform with that of Figure 7.

The N3^4−^ dynamics at room temperature in water solution is characterized, on the one hand, by the rigidity of the dcbpy ligands, due to the chelation to the Ru center and, on the other hand, by the flexibility of the NCS^−^ ligands, exploring conical-shaped regions (please see Figure 1, right panel, to recall the system under investigation). The vibrational dynamics induce therefore instantaneous deviations from the ideal $C_{2}$ symmetry, which could improve the transition probability of otherwise dark excited states [111]. The clustering procedure applied to the collected N3^4−^ trajectory suggested a partition into seven distinct clusters. Projection into the two-dimensional principal component subspace (actually accounting for 56.9% of the total variance) shows indeed a quite clear separation between the clusters and the medoids representing them (Figure 7). Again, the observed partial superposition could be a spurious effect of data visualization through a low-dimensional PCA. According to the feature values shown by the cluster medoids, these representative structures (reported in Figure 8) actually seem to capture both the conformational (torsional) freedom of the coordinated NCS^−^ ligands ($\phi_{1}$ and $\phi_{2}$ torsional angles) and the different degrees of asymmetry sampled by the N3^4−^ dynamics (Table 3, please refer also to Section 3.2 for N3^4−^ features definitions). In particular, the values of continuous symmetry measure of deviation from $C_{2}$ symmetry ($C_{2}$-CSM, Section 3.2) most sampled by the MD trajectory (distribution maxima at 0.09, 0.17, 0.22, 0.31, Figure 9) are close to the values by the cluster medoids, further confirming the representation capabilities of the latter.

Electronic absorption spectra of transition metal complexes are determined by several, closely spaced, excited states, differing in their spatial properties (i.e., metal and ligand-localized transitions, metal-to-ligand (ML) and ligand-to-metal (LM) charge-transfer (CT) transitions). From a practical point of view, this implies the computation (and characterization) of a high number of excited states with some level of theory to simulate the spectrum in a given energy range. Therefore, a clustering analysis performed on an MD trajectory (and so reducing the complete configuration dataset to a few, representative, structures) potentially appears even more convenient for the simulation and the interpretation of transition metal complex electronic spectra including finite-temperature effects.

**Figure 10:**
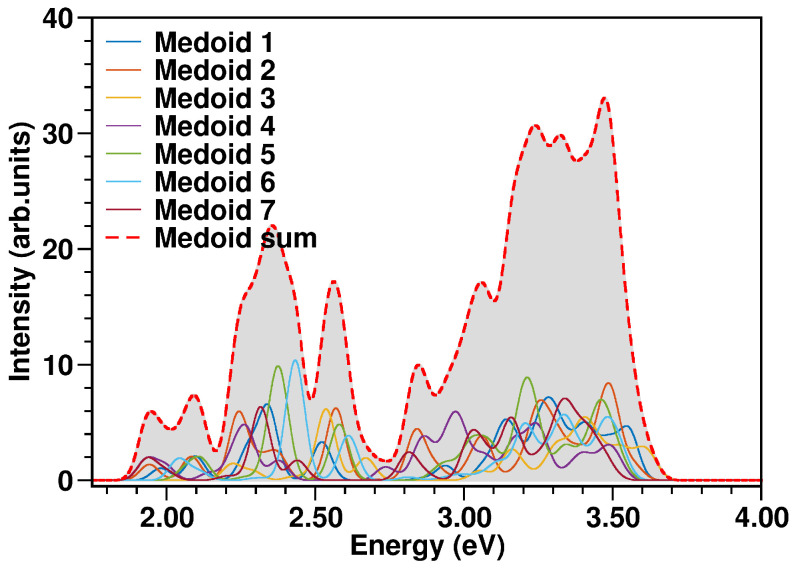
N3^4−^ absorption spectra (in eV) calculated at TD-B3LYP/C-PCM/def2-SVP/SDD(Ru) level of theory from each medoid, weighted by the population of the corresponding cluster and the spectrum resulting from the sum over the medoids (red dashed curve). The color code is presented in the graph legend. See Equation (5) and the procedure explained in Section 3.4 for more details.

**Figure 11:**
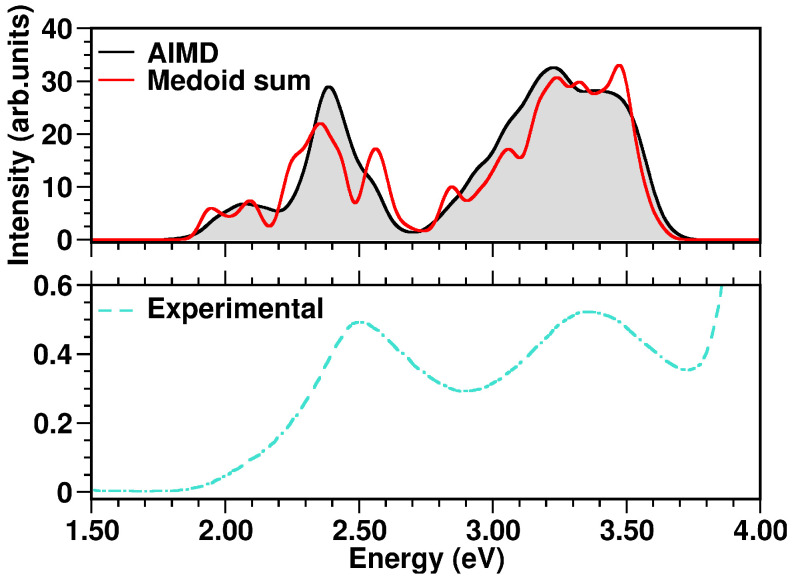
**Top** panel: comparison of N3^4−^ simulated absorption spectra in the 1.50–4.00 eV range. **Bottom** panel: experimental N3^4−^ UV-Vis spectrum, retrieved from Ref. [114], measured in water solution. The color code is presented in the graph legend.

The N3^4−^ electronic spectrum was simulated up to ∼3.7 eV, comprising the two experimentally characterized bands at ∼2.50 and ∼3.36 eV [114]. The spectra calculated for each cluster medoid actually slightly differ in the absorption band positions (energies) and intensities, since the medoids represent different regions of the accessible conformational space (Figure 10). In particular, the spectrum obtained from the only seven representative frames can actually quite well reproduce that from the complete MD sampling at T=298K in water solution (Figure 11), although with an increased sub-structure, due to the lower number of frames involved in the spectrum calculation. The selection of representative frames through a clustering analysis allowed one therefore to achieve a remarkable ∼70-fold decrease in the total computational cost for N3^4−^ electronic spectrum simulation.

Especially for transition metal complexes, the observed absorption bands can each be the result of many close transitions. Dataset reduction through a clustering analysis allowed an accurate N3^4−^ spectral characterization, which could be otherwise difficult to perform. In particular, the calculated band at 2.07 eV (Figure 11) results from medoid 1 S_2_, medoid 2 S_2_, medoid 7 S_1_, medoid 6 S_1_ and medoid 5 S_2_ states, which are mainly Ru → (dcbpy)_2_ ($\Omega_{RP}\approx 0.55$, $\Omega_{SP}\approx 0.25$, Table 4) CT states. Analogously, medoid 6 S_5_, medoid 7 S_5_, medoid 2 S_6_, medoid 1 S_5_ and medoid 3 S_5_, with similar metal-to-ligand charge-transfer (MLCT) spatial features, contribute to the more intense calculated band at 2.39 eV. The higher-energy bands are characterized instead by a less homogeneous set of excited states. In fact, the calculated band at 3.23 eV results from medoid 2 S_8_, medoid 7 S_13_ Ru → (dcbpy)_2_ states ($\Omega_{RP}\approx 0.55$, $\Omega_{SP}\approx 0.25$, Table 4), medoid 2 S_18_ Ru → (dcbpy)_2_ state, but with an increased dcbpy localized-excitation character ($\Omega_{RP}\approx 0.40$, $\Omega_{PP}\approx 0.30$), medoid 4 S_15_ Ru → (dcbpy)_2_ state, with increased (NCS)_2_ donor contribution ($\Omega_{RP}\approx 0.50$, $\Omega_{SP}\approx 0.40$) and medoid 7 S_19_ state, which is mainly an (NCS)_2_ → (dcbpy)_2_ CT state ($\Omega_{SP}\approx 0.40$, $\Omega_{RP}\approx 0.30$). The close calculated 3.38 eV band has instead a quite different average character. In fact, the contributing medoid 1 S_40_ state is mostly an (NCS)_2_ → (dcbpy)_2_ CT state ($\Omega_{SP}\approx 0.60$), medoid 2 S_37_ and medoid 5 S_33_ states have an increased localized character ($\Omega_{SP}\approx 0.40$, $\Omega_{PP}\approx 0.30$), while medoid 3 S_21_ and medoid 6 S_34_ are localized excitations on dcbpy ligands ($\Omega_{PP}\approx 0.60$ and ≈0.50, respectively).

## 3. Materials and Methods

### 3.1. Ab Initio Molecular Dynamics

The conformational flexibility of the TCNE:π:1ClN and N3^4−^ model systems were sampled through ab initio molecular dynamics simulations. In particular, the Atom-centered Density Matrix Propagation (ADMP) method was employed: the density matrix in an orthonormalized atomic basis is included in an extended Lagrangian as an additional degree of freedom and propagated together with the nuclear degrees, avoiding a self-consistent procedure at each step [115,116,117,118,119].

The TCNE:π:1ClN ground state trajectory was collected for 10 ps with a 0.2 fs time step, at the B3LYP/6-31G(d,p) [120,121,122] level of theory [35,94]. Temperature was kept at 298 K, through a velocity rescaling every 1 ps. Dichloromethane solvent effects were included through the conductor-like polarizable continuum model (C-PCM) [123,124,125,126,127,128]. Moreover, due to the π-stacked, non-covalent nature of the TCNE:1ClN complex, dispersion forces had to be modeled, employing Grimme’s correcting potential (GD3) [129,130,131,132,133,134].

The N3^4−^ system was simulated instead for 8.6 ps with a 0.1 fs time step [111]. A velocity rescaling every 1 ps allowed to keep a 298 K temperature. Explicit water solvation was included in the N3^4−^ ground state sampling, in order to better model the specific solute–solvent interactions at the several solvation sites. A 22 Å-radius spherical solvent box (∼1500 molecules) was extracted from a pre-equilibrated cubic one and placed around N3^4−^. A hybrid quantum mechanics/molecular mechanics potential was employed: B3LYP/def2-SVP [135] for the QM portion (the N3^4−^ molecule) with associated electronic core potential for the Ru atom [136] and the TIP3P water model [137] for the MM part (the water spherical box), re-parametrized to allow a bending motion [11]. The QM and MM potentials were combined through the ONIOM QM/MM scheme [138,139,140], including the MM charges into the QM hamiltonian (i.e., an “electronic embedding”). General AMBER Force Field [141] atom types (and so van der Waals non-bonding parameters) were assigned, moreover, to N3^4−^ atoms. Non-periodic boundary conditions were introduced through a hybrid explicit/implicit solvent model. Long-range electrostatic effects and short-range dispersion–repulsion forces between the explicit and the bulk solvent were, respectively, modeled through C-PCM self-reaction field and an empirical confining potential, which has to be parametrized for the specific solvent model [10,124,142,143,144]. We refer the reader to previous works for more details about the ab initio molecular dynamics simulations of the model systems and the employed potentials [34,94,111].

### 3.2. Feature Selection and Clustering of Molecular Dynamics Trajectories

Due to its large dimensions, it is often useful to transform the original dataset of the collected, *N*-frames long, trajectory (the configurations, i.e., the positions of each of the $N_{\mathrm{at}}$ atoms in the system, at each time step) into a matrix $X\in R^{N\times d}$, representing the data in some *d*-dimensional ($d\ll 3N_{\mathrm{at}}$) feature space, different from the coordinate space. The chosen features should adequately describe the properties of interest, without much loss of information [77]. Internal coordinates, such as bonds, angles and dihedrals or more specifically tailored parameters, according to the problem under study, can be employed as features.

In particular, the TCNE:π:1ClN trajectory was transformed into a feature space able to describe the orientation of the two molecular planes and the relative position of the two molecules, comprising the angle ($\left[0,180^{\circ }\right]$) between the versors normal to the TCNE and ClN planes, the versor representing the axis of rotation of the two planes (i.e., the versor orthogonal to the former ones) and the relative position vector (i.e., the vector between the two geometric centers). For N3^4−^, instead, a continuous symmetry measure of deviation from the ideal $C_{2}$ symmetry [145,146,147], calculated as the minimized root-mean-square deviation from the images generated through the $C_{2}$ symmetry operations, was considered. In particular, $C_{2}$-CSM was evaluated on the smallest subset of N3^4−^ atoms showing a symmetry not higher than $C_{2}$, as the complete molecule. Since the non-linearity of the NCS^−^ coordination in the water solution (C(NCS)-N(NCS)-Ru angle less than 180°) and their torsional mobility were previously recognized [111], the C(NCS)-N(NCS)-Ru-N(dcbpy) dihedrals describing the NCS^−^ orientations were also included. In this regard, to avoid problems due to the periodicity around ±180°, each dihedral ϕ was included as a (cos(ϕ),sin(ϕ)) pair to keep a metric feature space [148]. The MD datasets in the feature space X were standardized (i.e., shifted to zero mean and scaled to unit variance) before following analyses.

Clustering machine learning techniques allow one to partition a dataset, grouping similar instances according to a similarity measure, such as a metric (for instance, Euclidean) in the feature space [149]. Instances within a cluster should be similar to each other and different from those belonging to the other clusters. In K-Means [150] and K-Medoids [151,152,153] approaches, for a given number *K* of clusters, *K* cluster centers are obtained. The feature space is partitioned (tessellated) by assigning each instance to the closest center. The latter are found by minimization of a loss function, defined as the sum of the squared distances between each instance and the cluster center to which it is assigned:

(1) $$L\left(c_{k}\right)=\sum_{i}^{N}{\left\|x_{i}-c_{k}\right\|}^{2}$$

where $x_{i}$ belongs to the cluster *k*, $c_{k}$ is the corresponding center in the feature space and *N* is the number of “observations” (trajectory frames). While in the K-Means algorithm the cluster center is the mean of the cluster members and so does not have to correspond to any instance $x_{i}$ of the dataset, in the K-Medoids approach it is forced to be some $x_{i}$, such that the sum of the squared distances from the cluster members is the lowest (like a median).

MD trajectories of TCNE:π:1ClN and N3^4−^ model systems in their respective feature spaces were clusterized with the K-Medoids algorithm [152], since the cluster medoids, which are representative of the corresponding clusters, are trajectory frames themselves and, compared to K-Means centroids, should be less sensitive to possible outliers [151].

The optimal number of clusters *K* was chosen searching for an “elbow” (i.e., a slope change) in the plot of the inertia parameter (i.e., the minimized value of Equation (1)) as a function of *K* and evaluating the Calinski–Harabasz index [154], which is the ratio of between-cluster and within-cluster dispersions, being higher for a better clusterization into compact and separated clusters.

### 3.3. Dimensionality Reduction for MD Data Visualization

Principal component analysis (PCA) [155] is a popular dimensionality reduction technique. For some centered (i.e., zero-mean) data matrix X, its principal components $v_{j}$ are the eigenvectors of its covariance matrix C:

(2) $$C=\frac{1}{N}X^{T}X\;Cv_{j}=\lambda_{j}v_{j}$$

It can be shown that $v_{1}$ (the eigenvector corresponding to the largest eigenvalue $\lambda_{1}$) is the direction along which the variance of the data is highest, $v_{2}$ is the direction of highest variance in the subspace orthogonal to $v_{1}$, etc., while the eigenvalues $\lambda_{j}$ are the variance of the data along each $v_{j}$. Projection of the data on the subspace of the first $d_{r}\leq d$ principal components can be performed via the following:

(3) $$V=\left(v_{1}\cdots v_{d_{r}}\right)\;X_{r}=XV$$

where $\sum_{j}^{d_{r}}\lambda_{j}$ is the variance retained in the PC subspace.

PCA dimensionality reduction ($d_{r}=2$) of TCNE:π:1ClN and N3^4−^ trajectories was performed only for data visualization purposes on two-dimensional plots and not as a pre-processing step for clustering analysis. In fact, the dimensionalities of their respective feature spaces (Section 3.2) are actually quite small, likely not involving any “curse of dimensionality” issues.

### 3.4. Excited State Characterization and Spectra Simulations

TCNE:π:1ClN and N3^4−^ excited states were computed with the linear-response TD-DFT approach at CAM-B3LYP/GD3/C-PCM(DCM)/6-31+G(d,p) and B3LYP/C-PCM(water)/def2-SVP/SDD(Ru) levels of theory, respectively. Electronic spectra in the solution at T=298 K were simulated on 500 frame subsets of the collected MD samplings (i.e., every 20 fs and 17.2 fs, respectively). The first 8 and 40 singlet excited states were calculated for TCNE:π:1ClN and N3^4−^, respectively. The complete spectra were obtained by summation of Gaussian-shaped contributions over each frame and each calculated excited state:

(4) $$S_{li}\left(\nu \right)=f_{li}\;e^{-\frac{1}{2}{\left(\frac{\nu -\nu_{li}}{\sigma }\right)}^{2}}\;S\left(\nu \right)=\sum_{l}^{N_{\mathrm{fr}}}\sum_{i}^{N_{\mathrm{st}}}\;S_{li}\left(\nu \right)$$

where $f_{li}$ and $\nu_{li}$ are the oscillator strength and excitation energy of the *i*-th state of *l*-th frame and σ is a width parameter, set at $\sigma^{2}=0.001\;{\mathrm{eV}}^{2}$. Spectra estimated from the only cluster medoids were similarly calculated:

(5) $$S\left(\nu \right)=\sum_{k}^{K}p_{k}\sum_{i}^{N_{\mathrm{st}}}S_{ki}\left(\nu \right)$$

where *K* is the number of clusters and $p_{k}$ is the *k*-th cluster population.

Cluster medoid excited states were further characterized by fragment-based transition density Löwdin population analysis and related charge transfer descriptors, calculated with the TheoDORE package [156,157]:

(6) $$\begin{array}{c} \Omega_{AB}=\sum_{\mu \in A}\sum_{\nu \in B}{\left(S^{1/2}D^{0i}S^{1/2}\right)}_{\mu \nu }^{2} \end{array}$$

(7) $$\begin{array}{c} \omega_{\mathrm{CT}}=\frac{\sum_{A,B\neq A}\Omega_{AB}}{\sum_{A,B}\Omega_{AB}} \end{array}$$

where *A* and *B* are two molecular fragments and $D^{0i}$ is the transition density matrix for the S${}_{i}$ ← S_0_ excitation.

Ab initio molecular dynamics simulations and excited state calculations were performed with the Gaussian16 software package [158].

## 4. Conclusions

Unsupervised clustering methods have been employed as complementary approaches for an efficient exploration of the data resulting from MD simulations and electronic structure calculations. In this work, MD dataset reduction capabilities via unsupervised clustering techniques were applied for the ab initio modeling of electronic absorption spectra of the non-covalent charge-transfer TCNE:π:1ClN dimer and the [Ru(dcbpy)_2_(NCS)_2_]^4−^ complex in solution at room temperature. Cluster medoids, taken as representative structures, were found and analyzed in terms of main structural parameters, principal component dynamics, electronic excitations and charge transfer indices, showing how such medoids can satisfactorily cover the system dynamics and optical properties with a very good agreement with experiments.

The simulation of electronic absorption spectra usually demands expensive computations and requires dealing with the interplay of electronic excited states with the conformational freedom of the chromophores in complex matrices (i.e., solvents, biomolecules, crystals) at finite temperature. This work highlights the power of the unsupervised K-medoid clustering technique combined with a tailored selection of the feature space in reducing by ∼100 times the total cost of electronic and optical property computations on an MD sampling with no loss of accuracy and in preserving the molecular interpretation via the cluster medoids. In this regard, it could be very interesting to study how the medoids and the several conformational minima are related and this is a subject for further spectroscopic and weighting scheme developments.

## Figures and Tables

**Table 1:** Clustering feature values of the five cluster medoids from TCNE:π:1ClN trajectory. $\theta_{r}$: rotation angle (angle between versors normal to the two molecular planes, degrees), ${\hat{n}}_{r}$: rotation axis (versor normal to the former ones), ${\vec{r}}_{N-E}$: relative position vector (between 1ClN and TCNE geometric centers). Vector quantities are given as cartesian components (Å) in a fixed frame of reference.

Medoid	$\theta_{r}$	$n_{r,x}$	$n_{r,y}$	$n_{r,z}$	$r_{N-E,x}$	$r_{N-E,y}$	$r_{N-E,z}$
1	16.68	−0.660	0.398	0.638	1.513	2.847	2.190
2	14.60	−0.816	0.141	0.561	2.604	1.673	1.852
3	19.36	0.723	−0.407	−0.558	1.543	2.444	2.276
4	15.19	0.714	0.021	−0.699	2.666	1.773	1.736
5	16.27	0.112	−0.736	0.668	1.833	2.401	2.436

**Table 2:** Characterization of S_1_ and S_2_ excited states of TCNE:π:1ClN cluster medoids. $\nu_{i}$ (eV): vertical excitation energy, $f_{i}$: oscillator strength (arb. units), $\Omega_{AB}$: transition density population analysis for A (hole) and B (electron) fragments, $\omega_{\mathrm{CT}}$: charge transfer descriptor (please refer to Section 3.4 for definitions). Fragment labels: E: TCNE, N: 1ClN.

Medoid		$\nu_{i}$	$f_{i}$	$\Omega_{\mathrm{EE}}$	$\Omega_{\mathrm{EN}}$	$\Omega_{\mathrm{NE}}$	$\Omega_{\mathrm{NN}}$	$\omega_{\mathrm{CT}}$
1	$S_{1}$	1.807	0.002	0.014	0.000	0.968	0.018	0.968
	$S_{2}$	2.973	0.035	0.016	0.000	0.965	0.018	0.966
2	$S_{1}$	2.003	0.052	0.031	0.001	0.943	0.025	0.944
	$S_{2}$	2.835	0.014	0.018	0.001	0.955	0.027	0.955
3	$S_{1}$	1.928	0.026	0.019	0.000	0.963	0.017	0.963
	$S_{2}$	2.713	0.009	0.018	0.000	0.963	0.018	0.964
4	$S_{1}$	2.063	0.067	0.031	0.001	0.938	0.030	0.939
	$S_{2}$	2.863	0.013	0.014	0.000	0.953	0.032	0.954
5	$S_{1}$	2.084	0.005	0.009	0.000	0.980	0.012	0.980
	$S_{2}$	2.994	0.005	0.017	0.000	0.971	0.012	0.971

**Table 3:** Clustering feature values of the seven cluster medoids from N3^4−^ trajectory. $\phi_{1}$: C(NCS1)-N(NCS1)-Ru-N(dcbpy) dihedral angle (degrees), $\phi_{2}$: C(NCS2)-N(NCS2)-Ru-N(dcbpy) dihedral angle (degrees), $C_{2}$-CSM: continuous symmetry measure for deviation from $C_{2}$ symmetry.

Medoid	$\phi_{1}$	$\phi_{2}$	$C_{2}$-CSM
1	−30.77	5.57	0.176
2	−54.10	107.01	0.172
3	−143.43	140.61	0.219
4	52.26	−131.41	0.174
5	69.09	83.05	0.215
6	−127.10	40.02	0.116
7	107.85	−137.07	0.352

**Table 4:** Characterization of the excited states of N3^4−^ cluster medoids most contributing to the calculated absorption bands. $\nu_{i}$ (eV): vertical excitation energy, $f_{i}$: oscillator strength, $\Omega_{AB}$: transition density population analysis for A (hole) and B (electron) fragments, $\omega_{\mathrm{CT}}$: charge transfer descriptor (please refer to Section 3.4 for definitions). Fragment labels: S: (NCS)_2_, R: Ru, P: (dcbpy)_2_.

Medoid		$\nu_{i}$	$f_{i}$	$\Omega_{\mathrm{SP}}$	$\Omega_{\mathrm{RP}}$	$\Omega_{\mathrm{PP}}$	$\omega_{\mathrm{CT}}$
1	$S_{2}$	2.097	0.029	0.269	0.566	0.113	0.858
	$S_{5}$	2.342	0.086	0.284	0.542	0.119	0.851
	$S_{40}$	3.556	0.056	0.578	0.235	0.127	0.851
2	$S_{2}$	2.083	0.026	0.235	0.580	0.111	0.846
	$S_{6}$	2.569	0.079	0.323	0.507	0.103	0.862
	$S_{8}$	2.840	0.055	0.275	0.536	0.114	0.843
	$S_{18}$	3.237	0.046	0.265	0.422	0.271	0.713
	$S_{37}$	3.498	0.063	0.401	0.219	0.310	0.662
3	$S_{5}$	2.536	0.118	0.294	0.545	0.108	0.862
	$S_{21}$	3.389	0.043	0.133	0.215	0.583	0.391
4	$S_{15}$	2.983	0.056	0.366	0.511	0.087	0.894
5	$S_{2}$	2.114	0.022	0.276	0.572	0.086	0.876
	$S_{33}$	3.454	0.057	0.373	0.277	0.289	0.693
6	$S_{1}$	2.041	0.025	0.242	0.627	0.093	0.884
	$S_{5}$	2.433	0.141	0.267	0.570	0.109	0.859
	$S_{34}$	3.472	0.030	0.335	0.099	0.521	0.469
7	$S_{1}$	1.935	0.029	0.249	0.575	0.136	0.842
	$S_{5}$	2.316	0.101	0.267	0.551	0.121	0.844
	$S_{13}$	3.022	0.056	0.278	0.553	0.148	0.840
	$S_{19}$	3.165	0.059	0.427	0.312	0.212	0.773

## Data Availability

Not applicable.

## Abbreviations

The following abbreviations are used in this manuscript:

1ClN	1-chloronaphthalene
ADMP	Atom-centered density matrix propagation
C-PCM	Conductor-like polarizable continuum model
CT	Charge transfer
dcbpy	4,4′-dicarboxy-2,2′-bipyridine
DCM	dichloromethane
DFT	Density functional theory
LMCT	Ligand-to-metal charge-transfer
MD	Molecular dynamics
ML	Machine learning
MLCT	Metal-to-ligand charge-transfer
MM	Molecular mechanics
N3^4−^	[Ru(dcbpy)_2_(NCS)_2_]^4−^
PC	Principal component
PCA	Principal component analysis
PES	Potential energy surface
QM	Quantum mechanics
SDF	Spatial distribution function
TCNE	Tetracyanoethylene
TD-DFT	Time dependent density functional theory
